# Optical coherence tomography in secondary progressive multiple sclerosis: cross-sectional and longitudinal exploratory analysis from the MS-SMART randomised controlled trial

**DOI:** 10.1136/jnnp-2024-334801

**Published:** 2024-12-18

**Authors:** Floriana De Angelis, James R Cameron, Arman Eshaghi, Richard Parker, Peter Connick, Jonathan Stutters, Domenico Plantone, Anisha Doshi, Nevin John, Thomas Williams, Alberto Calvi, David MacManus, Frederik Barkhof, Siddharthan Chandran, Christopher J Weir, Ahmed Toosy, Jeremy Chataway, MS-SMART trial investigators, Jeremy Chataway

**Affiliations:** 1 Queen Square Multiple Sclerosis Centre, Department of Neuroinflammation, UCL Queen Square Institute of Neurology, Faculty of Brain Sciences, University College London, London, UK; 2 University College London Hospitals, Biomedical Research Centre, National Institute for Health and Care Research, London, UK; 3 The University of Edinburgh Centre for Clinical Brain Sciences, Edinburgh, Edinburgh, UK; 4 Edinburgh Clinical Trials Unit, The University of Edinburgh, Usher Institute of Population Health Sciences and Informatics, Edinburgh, UK; 5 Department of Neuroscience, University of Siena Faculty of Medicine and Surgery, Siena, Italy; 6 Department of Medicine, Monash University, Clayton, Victoria, Australia; 7 Advanced Imaging in Neuroimmunological Diseases lab (ImaginEM), Fundacio Clinic per la Recerca Biomedica, Barcelona, Spain; 8 Department of Radiology and Nuclear Medicine, VU University Medical Centre Amsterdam, Amsterdam, Noord-Holland, Netherlands

**Keywords:** MULTIPLE SCLEROSIS, IMAGE ANALYSIS, RANDOMISED TRIALS

## Abstract

**Background:**

Optical coherence tomography (OCT) inner retinal metrics reflect neurodegeneration in multiple sclerosis (MS). We explored OCT measures as biomarkers of disease severity in secondary progressive MS (SPMS).

**Methods:**

We investigated people with SPMS from the Multiple Sclerosis-Secondary Progressive Multi-Arm Randomisation Trial OCT substudy, analysing brain MRIs, clinical assessments and OCT at baseline and 96 weeks. We measured peripapillary retinal nerve fibre layer (pRNFL) and macular ganglion cell-inner plexiform layer (GCIPL) thicknesses. Statistical analysis included correlations, multivariable linear regressions and mixed-effects models.

**Results:**

Of the 212 participants recruited at baseline, 192 attended at 96 weeks follow-up. Baseline pRNFL and GCIPL thickness correlated with Symbol Digit Modalities Test (SDMT) (respectively, r=0.33 (95% CI 0.20 to 0.47); r=0.39 (0.26 to 0.51)) and deep grey matter volume (respectively, r=0.21 (0.07 to 0.35); r=0.28 (0.14 to 0.41)).

pRNFL was associated with Expanded Disability Status Scale (EDSS) score change (normalised beta (B)=−0.12 (−0.23 to −0.01)). Baseline pRNFL and GCIPL were associated with Timed 25-Foot Walk change (T25FW) (respectively, B=−0.14 (−0.25 to −0.03); B=−0.20 (−0.31 to −0.10)) and 96-week percentage brain volume change (respectively, B=0.14 (0.03 to 0.25); B=0.23 (0.12 to 0.34)). There were significant annualised thinning rates: pRNFL (−0.83 µm/year) and GCIPL (−0.37 µm/year).

**Conclusions:**

In our cohort of people with SPMS and long disease duration, OCT measures correlated with SDMT and deep grey matter volume at baseline; EDSS, T25FW and whole brain volume change at follow-up.

**WHAT IS ALREADY KNOWN ON THIS TOPIC**

- Many compounds may offer neuroprotection in progressive multiple sclerosis (MS); however, current outcome measures have limitations and are costly. Optical coherence tomography (OCT) has the potential to provide valuable and cost-effective outcome measures in progressive MS trials.

**WHAT THIS STUDY ADDS**

- Only a few trials have studied OCT measures longitudinally in progressive MS, especially in secondary progressive MS (SPMS) with long disease duration. Our study presents novel OCT data in a well-characterised SPMS cohort not on disease-modifying drugs.

**HOW THIS STUDY MIGHT AFFECT RESEARCH, PRACTICE OR POLICY**

- OCT may have a prognostic role in SPMS and could be used in cognitive studies or as a predictor of physical impairment.

## Introduction

 There are a large number of potential neuroprotective and remyelinating compounds in progressive multiple sclerosis (MS),^1^ but the current approach for testing new agents is time-consuming. Standard MRI-led phase 2b paradigms are expensive and require relatively long observation periods to detect meaningful change in relation to biological variability and slow accrual of atrophy.^2^ Hence, there is a need to develop more convenient non-MRI outcome measures to monitor disability progression in multisite clinical trials, and optical coherence tomography (OCT) is a clear candidate.

OCT uses low-coherence interferometry to obtain high-resolution images of the retina and its inner layers. Characteristic changes in the retina and optic nerve are found in most people with MS, regardless of their history of optic neuritis (ON), and are thought to reflect neuroaxonal loss.^3^,^6^ OCT retinal measures correlate with MS markers of inflammatory disease and neurodegeneration, as defined by relapses, new T2 lesions,^7 8^ clinical disability and MRI-derived brain atrophy.^9 10^ OCT measures that correlate most strongly are the peripapillary retinal nerve fibre layer (pRNFL) and macular ganglion cell-inner plexiform layer (GCIPL).

In recent times, OCT has gained an important role as a prognostic^11^,^15^ and diagnostic tool,^16^ easily transferable to clinical practice. However, the utility of OCT as an outcome measure in *progressive MS (PMS*) is still unclear. Additionally, most longitudinal studies in the field of OCT have included relapsing–remitting MS^912 15 17^,^19^ with limited application of OCT in progressive MS trials.^19^,^22^ Therefore, more longitudinal OCT clinical research in progressive MS is needed.

Here, we report a cross-sectional and longitudinal OCT substudy, embedded within the reported Multiple Sclerosis-Secondary Progressive Multi-Arm Randomisation Trial (MS-SMART), a phase 2b four-arm randomised controlled trial (RCT) investigating neuroprotection in secondary progressive MS (SPMS).^23 24^ The main trial results did not support any treatment effects for the three investigational agents (amiloride, fluoxetine and riluzole). The original preplanned OCT analysis also showed insufficient evidence for a difference in OCT measures between placebo and active arms overall.^24^ We report post hoc exploratory analyses that examine the value of pRNFL and GCIPL thickness as indicators of disease severity and progression in SPMS.

## Methods

We followed the Advised Protocol for OCT Study Terminology and Elements (APOSTEL) V.2.0 guidance for reporting quantitative OCT studies.^25^ We quality-checked OCT imaging based on the recommendations of the international consensus quality check criteria OSCAR-IB.^26^

### Study design and participants

The MS-SMART trial (NCT01910259) was a multicentre, phase 2b four-arm RCT investigating neuroprotection in people with SPMS in the United Kingdom.^23^ From January 2015 to June 2016, participants were randomised to receive amiloride, fluoxetine, riluzole or placebo for 96 weeks. Key inclusion criteria were age 25–65, Expanded Disability Status Scale (EDSS) score 4.0–6.5, evidence of disability progression in the preceding 2 years, and no MS disease-modifying therapy. SPMS was defined as a gradual worsening due to disease progression rather than relapses as a major cause of increasing disability in the preceding 2 years. People with relapses or treated with steroids within 3 months of their baseline visit were excluded. The primary outcome was MRI-derived brain atrophy at 96 weeks. The study found no significant differences between the active treatments and placebo.^23 24^

Participants from two MS-SMART trial centres (University College London (UCL) and the University of Edinburgh) were invited to an optional OCT study, for which we excluded those with non-MS ocular pathology or refractive errors beyond±6 dioptres. We obtained ocular pathology history from patient reports and available medical or ophthalmic records. We also excluded patients if we found evidence of unexpected non-MS retinal pathology on OCT. Medical histories of visual symptoms and previous ON were obtained from patient reports and records. For patients with indeterminate history of ON, we used p-RNFL normative data to infer previous ON according to age groups (18–29, 30–39, 40–49, 50–59, 60–69 year of age groups) using the reference table from Kennedy *et al* assuming a 99% CI.^27^ Additionally, subclinical ON was assumed when pRNFL inter-eye thickness difference exceeded 20%.^15^

### Standard protocol approvals, registrations and patient consents

The MS-SMART trial adhered to the Declaration of Helsinki and ICH Good Clinical Practice guidelines. Ethics approval was granted (REC ID: 13/SS/0007), and all patients provided written informed consent. Additional consent was obtained for the OCT substudy.

#### Outcomes

All participants underwent OCT scanning for pRNFL and GCIPL thickness, clinical assessment and brain MRI at baseline and 96 weeks. Clinical measures included EDSS, 9-hole peg test (9HPT), Timed 25-Foot Walk (T25FW), Symbol Digit Modalities Test (SDMT) and Sloan low contrast letter acuity (LCLA) 2.5% charts. MRI measures included T2 lesion volume, normalised whole-brain volume (WBV) and grey matter volume (cortical grey matter volume (CGMV) and deep grey matter volume (DGMV)) at baseline and 96 weeks, and percentage brain volume change (PBVC) from baseline to week 96.

#### OCT scanning and analysis

We (FDA, JRC, DP) performed OCT imaging on a spectral domain OCT Spectralis device (Heyex V.6.9.4.0, Heidelberg Engineering, Heidelberg, Germany) at the two study sites, without pupillary dilatation. Ambient light was dimmed. Two scans per eye were acquired: (1) pRNFL circular scan (diameter 12°, 1536 A-scans, 1 B-scan, automatic real time (ART) 100) manually centred on the optic nerve head with enabled eye-tracking modality. (2) Macular volume scans (UCL site settings: 20°×20° volume scan, 25 B-scans, 1024 A-scans per B-scan, vertical alignment, ART 9; University of Edinburgh site settings: 30°×25° volume scans, 61 B-scans, 768 A-scans per B-scan, posterior pole alignment, ART 12) centred about the fovea with eye tracking enabled. For longitudinal imaging registration, the ‘follow-up option’ was selected. We extracted macular thickness values using a thickness map on a 3 mm-diameter Early Treatment Diabetic Retinopathy Study grid. We obtained macular layer segmentation to quantify the GCIPL thickness with automated segmentation software provided by the vendor (Spectralis, V.6.8a, Heidelberg Engineering, Heidelberg, Germany). OCT imaging and retinal layer segmentation were visually inspected. We (FDA, JRC) manually corrected major segmentation errors and rejected scans that violated international consensus criteria.^26^ FDA and JRC were blind to participant’s clinical and radiological data at the time of quality check, manual segmentation or manual correction of automated segmentation errors to address potential bias.

#### MRI scanning and analysis

Brain MRI scans were obtained on a 3T Philips Achieva scanner (Philips Healthcare, Best, the Netherlands) at the UCL site and on a 3T Siemens Verio scanner (Siemens Healthcare, Erlangen, Germany) at the University of Edinburgh site. We acquired the following scans: axial proton density (PD)-weighted fast spin echo, in-plane resolution 1 mm^2^, slice thickness 3 mm; axial T2-weighted fast spin echo, in-plane resolution 1 mm^2^, slice thickness 3 mm; axial fluid-attenuated inversion recovery, in-plane resolution 1 mm^2^, slice thickness 3 mm; axial three-dimensional (3D)-T1 spoiled gradient-recalled echo with 1 mm^3^ resolution. MRI scan quality control was performed at the central MRI facility (Queen Square MS Centre Trial Office).

To quantify T2 lesion volume at baseline, we manually contoured lesions on PD/T2 scans using a semi-automated tool (JIM7, Xinapse Systems). T2 lesion masks were used to lesion-fill 3D T1-weighted images, and brain images were extracted to obtain WBV, grey matter volume (subsegmented into CGMV and DGMV), white-matter volume and total intracranial volume using the Geodesic Information Flows algorithm.^28^ Scaling factors for brain volume measures normalised against head size were estimated with Structural Image Evaluation, using Normalization, of Atrophy - Cross-sectional (SIENAX).^29 30^ For longitudinal analysis, we estimated PBVC with Structural Image Evaluation, using Normalization, of Atrophy (SIENA).^24 29 30^

#### Statistical analysis

All statistical analyses were performed with R, V.4.4.1 (R Foundation for Statistical Computing).^31^ We describe quantitative variables using means and SD or medians and IQRs, and ordinal variables with absolute numbers and percentages.

As the trial included four agents (amiloride, fluoxetine, riluzole and placebo), all statistical models were adjusted for treatment arm, to account for any potential treatment effects during the trial period. Additionally, due to OCT scanning differences between UCL and Edinburgh, all statistical models were also adjusted by site. Finally, all statistical models were adjusted for history of ON at the eye or patient level as appropriate.

### Baseline analysis

We used Pearson’s or Spearman’s correlations as appropriate to look at associations between baseline OCT, MRI and clinical variables. This analysis was carried out at the patient level; therefore, to obtain a unique pRNFL and GCIPL value per eye, we averaged the OCT and LCLA 2.5% values across the eyes for patients with no history of ON and used only the values from the unaffected eye for those without previous history of ON. We removed patients with history of bilateral ON.

### Longitudinal analysis

Statistical models were estimated separately for pRNFL and GCIPL as response variables.

To evaluate annualised OCT rates of change, we used linear mixed-effects models at eye-level accounting for within-patient and between-visit inter-eye measures (with random subject and eye-specific intercepts and random slopes in time). The fixed effects interactions allowed us to evaluate differences in rates of change between ON and non-ON eyes. Models were adjusted for age, sex, disease duration, site, treatment allocation.

To examine the associations between baseline OCT measures and clinical/MRI interval changes after 96 weeks, we used linear mixed-effects models at the eye level (subjects as random intercepts), adjusted for age, sex, disease duration, site, treatment allocation, ON history. As visual acuity can affect SDMT performance, we also adjusted for low contrast visual acuity as appropriate.

To analyse the relationship between OCT measure thickness percentage change and brain volume percentage change, we performed multivariable linear regressions adjusting for age, sex, disease duration, site, treatment allocation and history of ON. This analysis was done at the patient level, removing eyes with previous ON. To achieve that, we first calculated the pRNFL and GCIPL percentage thickness change per each unaffected eye as follows:

{[(96weekOCTmeasure−baselineOCTmeasure)÷baselineOCTmeasure]×100}. Subsequently, we averaged the OCT percentage thickness change across both eyes, to obtain only one value per subject.

We considered p<0.05 to be statistically significant. We did not impute missing values. Multiple comparison corrections were not performed due to the exploratory nature of this study.

## Results

### Baseline analysis

#### Baseline characteristics

**Figure 1:**
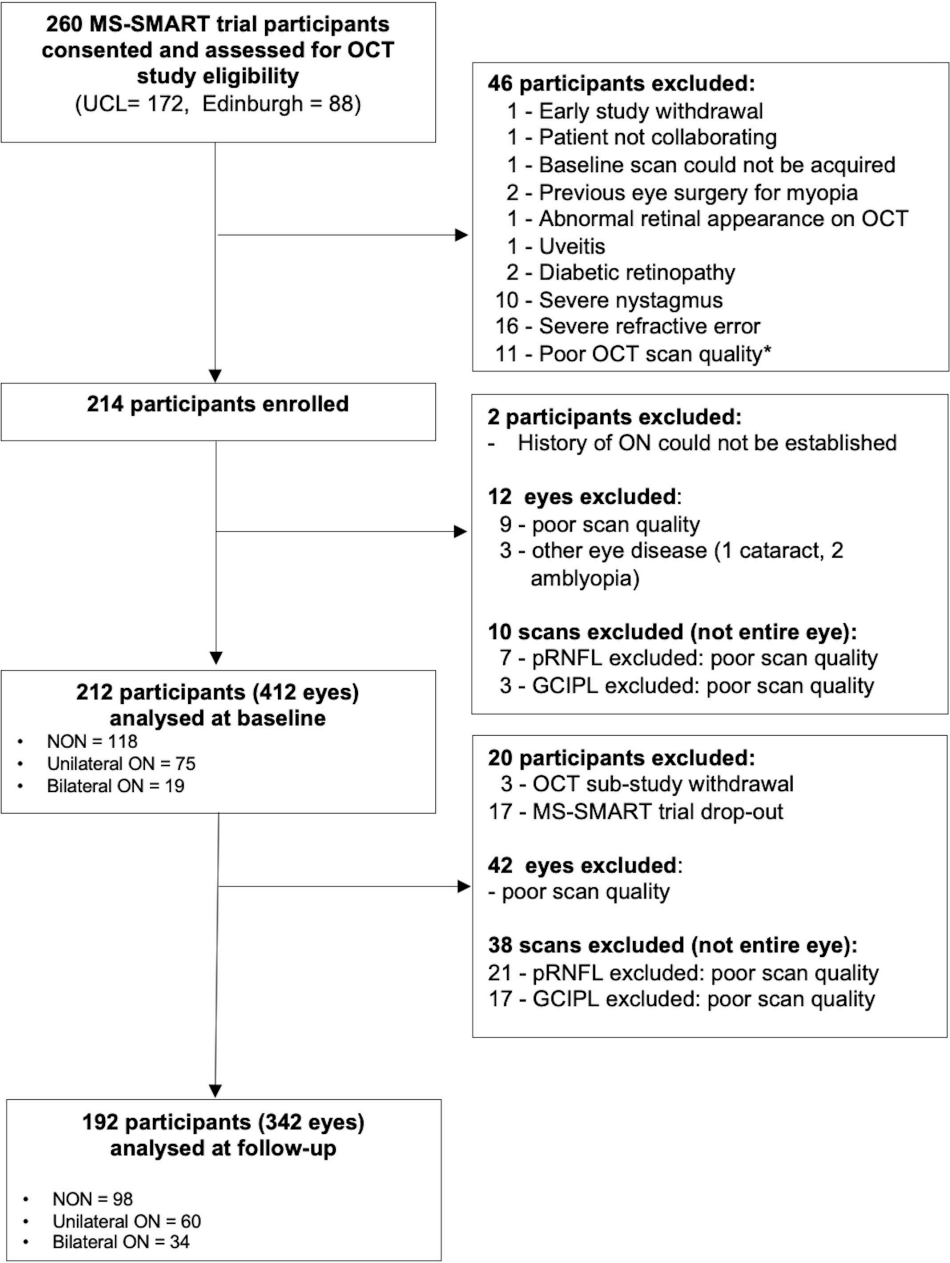
Study profile. Total number of MS-SMART trial participants randomised at UCL= 176 (172 consented the OCT substudy). Total number of participants randomised at Edinburgh = 93 (88 consented the OCT substudy). *For n=11 subjects excluded due to poor OCT imaging quality possible underlying retinal abnormalities were not systematically recorded. GCIPL, ganglion cell-inner plexiform layer; NON, no history of optic neuritis; OCT, optical coherence tomography; ON, optic neuritis; pRNFL, peripapillary retinal nerve fibre layer; UCL, University College London.

Of the total 269 patients enrolled in the MS-SMART trial at the UCL and University of Edinburgh centres, 260 consented to take part in the OCT substudy. Of these, 214 ultimately underwent OCT scanning at baseline (figure 1). After quality checking of baseline scans, 13 eyes were excluded. Additionally, two subjects were excluded from the analysis as we could not determine if they had previously experienced ON. For 17 subjects, a history of ON was inferred using absolute pRNFL thickness thresholds based on normative data (n=6 classified as NON, n=8 as bilateral ON and n=3 as unilateral ON). Six subjects were classified as having subclinical unilateral ON based on >20% pRNFL thickness difference between the two eyes. Details of the study profile are shown in figure 1.

Table 1 reports the baseline characteristics. Participants had average age of 54 years, had a long mean disease duration (around 22 years) and severe disability (median EDSS 6.0). Most of the participants had a disease duration longer than 5 years (97%), with a median disease duration of 21 years (IQR 16–29 years).

**Table 1:** Baseline characteristics of participants

	n=212
**Age,** years mean (SD)	54.4 (7)
**Female sex,** number **(**%)	149 (63)
**Disease duration,** years mean (SD) median (IQR)	22.3 (9.8) 21 (16–29)
**Progression duration,** years mean (SD)	8.2 (5.8)
**EDSS,** score median (IQR)	6.0 (5.5–6.5)
**9HPT**, sec mean (SD)	39.8 (53.7)
**T25FW,** sec mean (SD)	18.5 (22.9)
**SDMT,** number of correct answers median (IQR)	48 (39–53)
**LCLA 2.5%,** number of correct answers median (IQR)	18 (10–28.125)
**pRNFL** thickness, µm mean (SD)	83.3 (13.4)
**GCIPL** thickness, µm mean (SD)	75.2 (13.9)
**WBV,** mL mean (SD)	1426.9 (75.1)
**DGMV,** mL mean (SD)	45.2 (3.8)
**CGMV,** mL mean (SD)	797.4 (39.4)
**T2LV,** mL mean (SD)	11.9 (11.4)

9HPT is calculated as the average of the mean of the two attempts with each hand. OCT and LCLA measures are calculated as right- and left-eye value means.

CGMV, cortical grey matter volume; DGMV, deep grey matter volume; EDSS, Expanded Disability Status Scale; GCIPL, ganglion cell-inner plexiform layer; 9HPT, 9-hole peg test; LCLA, low contrast letter acuity; pRNFL, peripapillary retinal nerve fibre layer; SDMT, symbol digit modalities test; T25FW, Timed 25-Foot Walk; T2LV, T2 lesion volume; WBV, whole-brain volume.

There were no substantial differences in participant characteristics between subjects that consented or not to take part in the OCT study or across the two study sites, but the UCL participants appeared to be slightly less impaired on EDSS and SDMT measures (online supplemental tables 1 and 2).

### Correlation analyses

Results from the baseline correlation analysis are summarised in table 2.

The pRNFL and GCIPL thicknesses strongly correlated with visual acuity as measured by LCLA 2.5%. OCT measures were not correlated with baseline EDSS, T25FW or 9HPT values. However, they did show significant associations with the cognitive measure SDMT (table 2).

**Table 2:** Correlation analysis between OCT and clinical and MRI measures at baseline

		pRNFL	GCIPL
**Clinical variables**			
**EDSS,** score	*r*	−0.08	−0.12
*CI*	−0.22 to 0.06	−0.25 to 0.03
**9HPT,** s	*r*	0.03	0.03
	*CI*	−0.11 to 0.17	−0.11 to 0.17
**T25FW, s**	*r*	0.02	−0.02
	*CI*	−0.12 to 0.16	−0.16 to 0.12
**SDMT,** number of correct answers	*r*	**0.33**	**0.39**
	*CI*	0.20 to 0.47	0.26 to 0.51
**LCLA 2.5%,** number of correct answers	*r*	**0.45**	**0.48**
	*CI*	0.33 to 0.58	0.37 to 0.60
**MRI variables**			
**WBV,** mL	*r*	0.16	0.15
	*CI*	0.02 to 0.30	0.01 to 0.29
**DGMV,** mL	*r*	**0.21**	**0.28**
	*CI*	0.07 to 0.35	0.14 to 0.41
**CGMV,** mL	*r*	0.11	0.11
	*CI*	−0.03 to 0.25	−0.03 to 0.25
**T2LV,** mL	*r*	−0.16	**−0.24**
	*CI*	−0.30 to −0.02	−0.37 to −0.10

Eyes with history of optic neuritis were removed (number of subjects analysed=193). Person’s or Spearman’s correlations were used according to the variable nature (continuous or categorical, respectively). Correlation coefficients of magnitude below 0.2 (very weak) were considered non-significant.

CGMV, cortical grey matter volume; DGMV, deep grey matter volume; EDSS, Expanded Disability Status Scale; GCIPL, ganglion cell inner-plexiform layer; 9HPT, 9-hole peg test; LCLA, low contrast letter acuity; pRNFL, peripapillary retinal nerve fibre layer; SDMT, symbol digit modalities test; T2LV, T2 lesion volume; WBV, whole-brain volume.

Additionally, thicker retinal measures (pRNFL and GCIPL) correlated with larger DGMV. GCIPL only correlated with smaller T2LV.

No other significant correlations were noted between OCT and MRI measures.

### Longitudinal analysis

#### Annualised change of OCT measures

**Figure 2:**
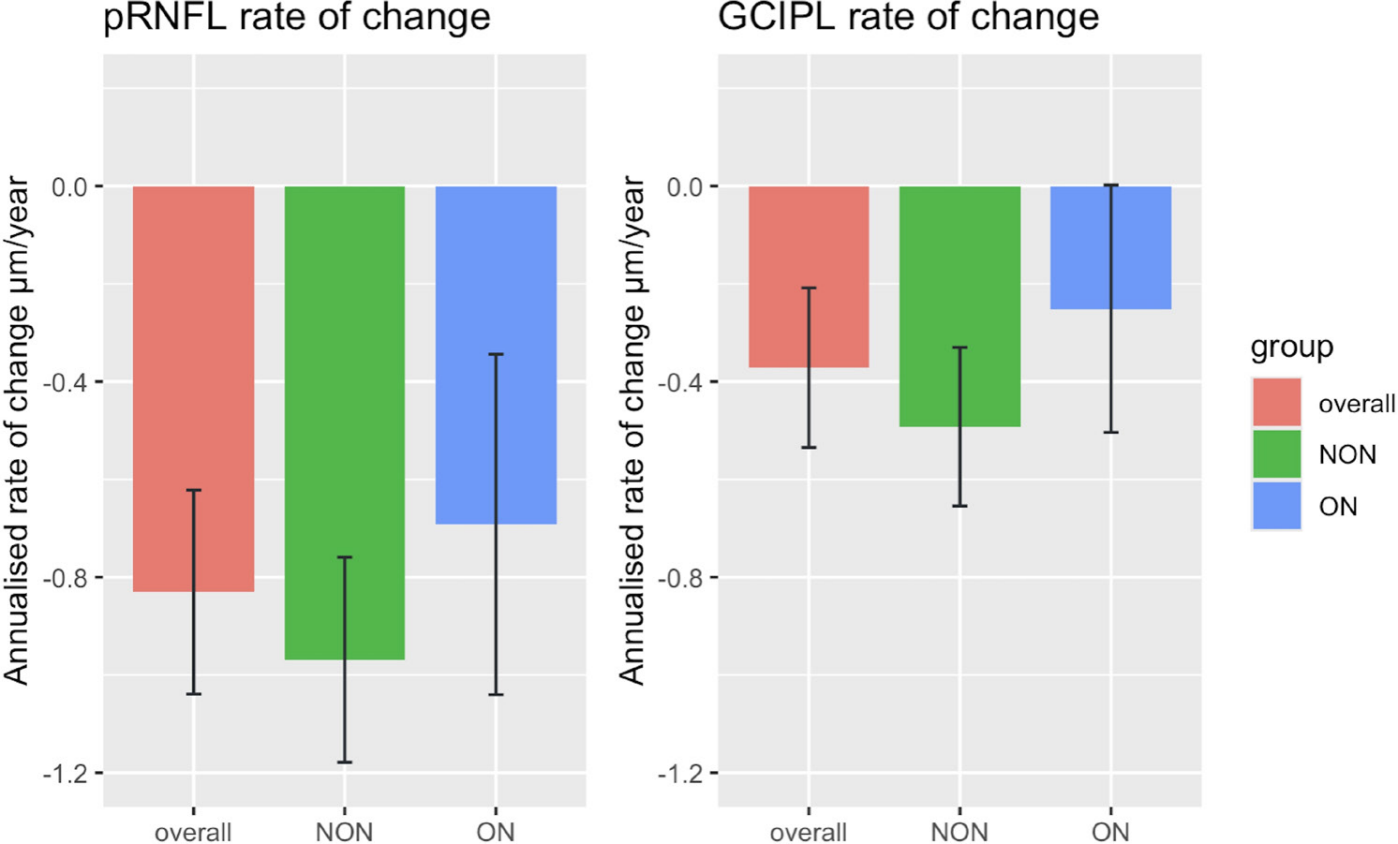
. Annualised rates of change for pRNFL and GCIPL. Mixed effect models are analysed at the eye level adjusted for history of optic neuritis. The bars represent annual mean changes in thickness (µm). Error bars represent confidence intervals. Eye are grouped as: overall group (including all eyes regardless history of optic neuritis); ON group (only eyes with history of optic neuritis), NON group (only eyes without history of optic neuritis). GCIPL, ganglion cell-inner plexiform layer; NON, no history of optic neuritis; ON, optic neuritis; pRNFL, peripapillary retinal nerve fibre layer.

Figure 2 shows the annualised rates of change of pRNFL and GCIPL in the overall group including all eyes regardless history of ON, with statistically significant thinning demonstrated by mean values of −0.83 µm/year (95% CI −1.04 to −0.62) and −0.37 µm/year (95% CI −0.53 to −0.21), respectively (figure 2). Mean changes of thinning when looking at eyes with history ON were −0.69 µm/year (95% CI −1.04 to −0.34) for pRNFL and −0.25 µm/year (95% CI −0.50 to 0.002) for GCIPL, compared with mean changes of −0.97 µm/year (95% CI −1.18 to −0.76) and −0.49 µm/year (95% CI −0.65 to −0.33) for eyes without history of ON (figure 2). There was no statistically significant difference in the rate of thinning between the groups based on ON history. There was no statistically significant association between disease duration and retinal layer thinning in the mixed effect models (CI −0.28 to 0.09 for the relationship between pRNFL and disease duration; CI −0.36 to 0.04 for the relationship between GCIPL and disease duration).

As the GCIPL acquisition protocols differed between the two sites, we conducted an additional analysis focusing on the rate of GCIPL change at each study site separately (online supplemental fig. 1 and online supplemental table 5**–**7). This analysis indicated that the overall study’s GCIPL thinning rate was primarily influenced by the UCL site, where the rate of change was −0.23 µm/year (95% CI −0.38 to −0.08), compared with Edinburgh, where the rate of change was −0.76 µm/year (95% CI −1.86 to −0.34). However, the number of GCIPL scans available at the UCL site (n=231) was more than double that of Edinburgh (n=94).

#### Associations between baseline OCT measures and clinical and MRI change

Significant associations were found between thinner baseline pRNFLs and GCIPLs and greater clinical disability worsening and/or thinner brain volumes. Table 3 shows that thinner pRNFL at baseline was associated with greater disability as measured by EDSS after 96 weeks. Larger pRNFL and GCIPL at baseline were associated with slower T25FW speed at follow-up. Also, larger baseline OCT measures were associated with reduced rates of whole brain atrophy (PBVC) at 96 weeks. No associations were seen between OCT measures and changes in 9HPT, SDMT or other MRI variables.

**Table 3:** Baseline OCT measures associated with changes of clinical variables over 96 weeks

		Variable change between baseline and week 96						
**Predictor variable at baseline**		**EDSS** score	**9HPT** sec	**T25FW** sec	**SDMT** correct answers	**PBVC** %	**DGMV** mm^3^	**CGMV** mm^3^
**pRNFL,** µm	*β*	**−0.12**	0.04	**−0.14**	0.05	**0.14**	−0.003	−0.03
	*CI*	**−0.23** **−0.01**	−0.08 0.17	**−0.25** **−0.03**	−0.08 0.17	**0.03** **0.25**	−0.11 0.11	−0.14 0.08
**GCIPL,** µm	*β*	−0.11	0.07	**−0.20**	0.07	**0.23**	−0.01	0.04
	*CI*	−0.22 0.003	−0.06 0.20	**−0.31** **−0.10**	−0.06 0.20	**0.12** **0.34**	−0.13 0.10	−0.07 0.15

The data presented are normalised.

Mixed-effect models adjusted for age, sex, disease duration, trial site, history of optic neuritis and treatment allocation. When 9HPT and SDMT were the predictors, models were also adjusted for visual acuity.

CGMV, cortical grey matter volume; DGMV, deep grey matter volume; EDSS, Expanded Disability Status Scale; GCIPL, ganglion cell-inner plexiform layer; 9HPT, 9-hole peg test; PBVC, percentage brain volume change; pRNFL, peripapillary retinal nerve fibre layer; SDMT, symbol digit modalities test; T25FW, Timed 25-Foot Walk; WBV, whole-brain volume. ; β, standardised regression coefficient from mixed-effects model.

#### Relationship between percentage OCT retinal thickness change and PBVC over 96 weeks

pRNFL mean (SD) percentage thickness change was −2.00% (2.82), calculated at the patient level as the average between left and right eye. There was no significant association between percentage thickness change of pRNFL and PBVC (β=0.07%, 95% CI −0.22 to 0.37).

GCIPL mean (SD) percentage thickness change was −1.11% (2.17). The associations between GCIPL percentage thickness change and PBVC was statistically significant (β=0.25%, 95% CI 0.01 to 0.50).

## Discussion

We report a large prospective longitudinal OCT study carried out within an RCT of people with SPMS using a Heidelberg Spectralis device. We observed significant mean annualised retinal thinning of −0.83 µm/year for pRNFL and −0.37 µm/year for GCIPL. This indicates that OCT can detect retinal changes even in patients with long-standing MS of over 20 years, as highlighted in a recent study.^32^ However, we also noticed that the retinal layer thinning was more evident in the unaffected eyes, suggesting that, after more than 20 years from MS onset, there may be a floor effect in retinal thinning, especially in eyes that previously experienced ON, as found elsewhere.^33^ Furthermore, this floor effect appears to affect the GCIPL more than the pRNFL. We also found that OCT measures, and especially GCIPL, are significantly associated with information processing speed and DGMVs at baseline, in support of previous research suggesting that OCT changes may reflect a loss of higher brain functions and overall neurodegeneration.^5 34^ Although OCT did not correlate with the most common MS disability measures at baseline, both baseline pRNFL and GCIPL thickness appeared to have prognostic value, as they were associated with increased disease severity (EDSS and walking speed worsening) and brain volume loss (PBVC).

OCT has been used as an outcome measure in only a few RCTs of people with progressive MS to date.

In the lipoic acid phase 2 study in SPMS,^21 35^ non-statistically significant mean pRNFL and GCIPL atrophy rates were found, and there were no correlations between OCT changes and whole-brain or grey matter atrophy. Our participants had higher baseline pRNFL and GCIPL mean thicknesses (around 83 µm and 75 µm, respectively), compared with those in the lipoic acid study (77 µm and 67 µm, respectively). Additionally, our sample size was larger, participants were younger and had shorter disease duration (22 vs 30 years).^21^

The OCT substudy of the SPRINT-MS trial (ibudilast vs placebo)^22 36 37^ did not report significant pRNFL thinning. However, they used different OCT devices (Zeiss Cirrus vs Heidelberg Spectralis) and included people with PPMS, with an overall shorter disease duration (approximately half that in MS-SMART), which could account for different changes in OCT measures over time. Although PPMS and SPMS share similar neuropathological mechanisms, they may differ clinically.^38^

We found an association between baseline OCT measures and DGMV at baseline. In a study that included people with progressive MS, Saidha *et al* found that both pRNFL and GCIPL thicknesses were significantly associated with CGMV.^39^ The same research group carried out a 4-year longitudinal study and suggested that, over time, GCIPL loss mirrors whole-brain and grey matter atrophy, especially in people with progressive MS, thereby reflecting underlying disease progression.^5^

We also found that baseline OCT measures and GCIPL percentage thickness change, and not pRNFL, were associated with PBVC after 96 weeks, which is in keeping with previous reports suggesting that GCIPL may reflect global neurodegeneration better than pRNFL.^5^

Regarding disability, we did not find significant associations between OCT measures and EDSS at baseline, likely due to the narrow EDSS range at baseline. However, the longitudinal data showed that baseline pRNFL measure could predict EDSS changes, consistently with other studies.^14 15^ Additionally, both baseline GCIPL and pRNFL were inversely associated with worsening in mobility (T25FW), suggesting that retinal thinning may reflect clinical worsening in SPMS.

In the cross-sectional analyses, pRNFL and GCIPL thickness were associated with SDMT. Already in 2013, Wieder *et al* found that information processing speed measured with PASAT and SDMT and visual function tests were correlated in relapsing MS.^40^ However, there is a paucity of studies investigating the relationship between OCT and cognition in MS. Recently, Alba-Arbalat and colleagues found that a strong relationship between the decrease in retinal thickness in people with MS and cognitive decline throughout the course of the disease, with a more pronounced effect observed after 5 years from disease onset.^41^ Baetge *et al* analysed 50 subjects with MS (n=44 RRMS, n=6 SPMS) and found that OCT-derived retinal measures were correlated with cognitive flexibility but not with information processing speed (SDMT). However, the study participants had mild to moderate disability (mean EDSS 2.59) and relative short disease duration (median about 7 years).^42^ Coric *et al* found that atrophy of the pRNFL and GCIPL was significantly associated with increased odds of being cognitively impaired in a group of 217 people with MS (SPMS 26%, PPMS 13%).^34^ Bsteh *et al* found that OCT measures were predictors of cognitive decline.^14^ In our study, the associations between OCT measures and SDMT were not maintained in the longitudinal analyses, suggesting that retinal atrophy occurs more rapidly than the development of an established processing speed deficit.

Our study has several limitations. We lacked a healthy control group, which prevented us from ruling out normal ageing as the cause of the retinal thinning observed, rather than MS-related atrophy. To account for the effect of ageing on retinal atrophy, we adjusted all our statistical models for age. As reference, the IMSVISUAL consortium recently reported normative data from healthy volunteers, showing a decline of −1.31 µm in pRNFL and −1.05 µm in GCIPL per decade.^27^ The rate of thinning we observed for GCIPL (−0.37 µm/year) is in keeping with other studies, which suggest thinning rates in people with MS ranging from −0.30 to −0.55 µm/year, compared with −0.20 µm/year in healthy controls.^19^ In contrast, the rate of pRNFL annualised atrophy in our study (−0.83 µm/year) was only slightly lower than rates described in other investigations, which have reported annual thinning rates of 1–2 µm/year in people with MS. However, compared with GCIPL, pRNFL thinning has shown greater variability across different studies.^19^

The macular OCT acquisition protocols, and specifically the number of B scans, were not the same between the two study sites (UCL and Edinburgh), which we need to keep in mind when interpreting the results. The GCIPL has been repeatedly shown to be superior to the pRNFL as a measure of neurodegeneration, with high reproducibility. Using a small number of B scans, like in the UCL OCT protocol, we may have underestimated the change of GCIPL over time. In addition, the observed mean (SD) thickness percentage changes in pRNFL and GCIPL after 96 weeks were less than 2%, reflecting a small effect size. We speculate that this may be due to floor effect^33^; however, the acquisition parameters need to be kept in mind. Advances in OCT acquisition and processing could at least in part reduce this variability in future studies. However, mindful of the different GCIPL acquisition protocols between UCL and Edinburgh, we adjusted all the statistical models by site.

Another limitation of our study was the potential effects of drugs with putative neuroprotective effects in the treatment arms of the MS-SMART clinical trial, which may have had unknown effects on our OCT measurements. However, the main trial results did not show any neuroprotective effects of amiloride, fluoxetine or riluzole on brain atrophy measures. Additionally, we adjusted all statistical analyses for trial arms.

In conclusion, our study suggests that, despite long disease duration, pRNFL and GCIPL thinning is detectable over a 96-week period and is likely to reflect ongoing neuroaxonal loss (neurodegeneration), but there seem to be a floor effect in the retinal thinning especially for the GCIPL and for eyes with previous ON. This is supported by the observed association between OCT measures, grey matter volume and PBVC. OCT measures correlated significantly with information processing speed measure (SDMT) in cross-sectional analyses and were predictors of clinical worsening (EDSS and T25FW) after 96 weeks. These findings may help inform future research utilising OCT measures in people with SPMS.

## Supplementary material

10.1136/jnnp-2024-334801 online supplemental file 1

## Data Availability

Data are available upon reasonable request.
