# Supplementary material for: Optical coherence tomography in secondary progressive multiple sclerosis: cross-sectional and longitudinal exploratory analysis from the MS-SMART randomised controlled trial
Source: J Neurol Neurosurg Psychiatry. 2024 Dec 18;96(7):e334801. doi: 10.1136/jnnp-2024-334801 (PMC12322454; doi:10.1136/jnnp-2024-334801)
Supplement: online supplemental file 1 [file jnnp-96-7-s001.pdf]

Supplementary Material

Supplementary Figure 1. Rate of change of GCIPL across the two sites.

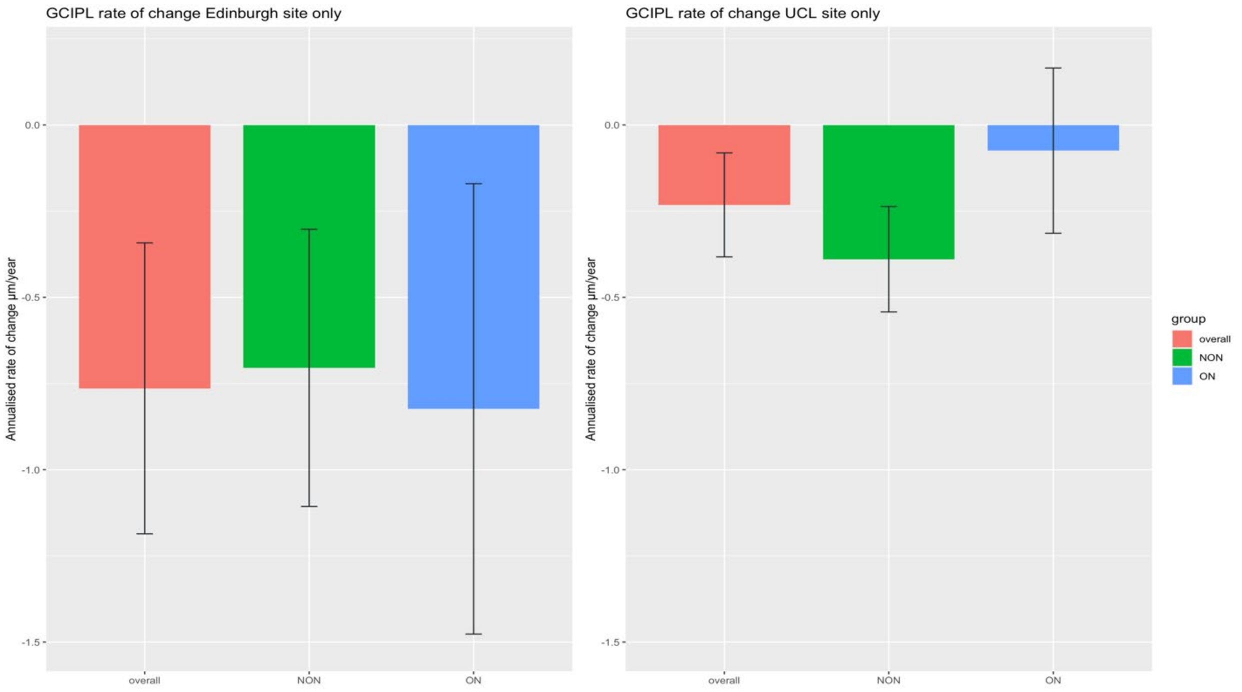

The error bars represent 95% confidence intervals.
GCIPL available for longitudinal analyses at week 96:
Edinburgh site n= 94
UCL site n= 231

Supplementary Table 1. Baseline characteristics of subjects that did not consent to take part in the OCT study versus those who consented.

	Not consented n=9	Consented n=260	P value
Age, *years mean (SD)*	55.5 (8.9)	54.4 (7.3)	0.720
Female sex, *no (%)*	9 (100)	190 (73)	-
Disease duration, *years mean (SD)*	22.9 (11)	22.3 (9.7)	0.872
Progression duration, *years mean (SD)*	6.5 (4.1)	8.4 (6)	0.210
EDSS, *score^ median (IQR)*	6.5 (6.5-6.5)	6.0 (6.0–6.5)	**0.012**
9HPT*, sec mean (SD)*	92.4 (130.6)	44.2 (62.9)	0.301
T25FW, *sec mean (SD)*	35.1 (38.3)	19.7 (27.3)	0.263
SDMT, *no. of correct answers median (IQR)*	34 (29-46)	48 (37-52)	0.053
SLCVA 2.5%, *no. of correct answers median (IQR)*	5 (1.125-13.375)	17 (8.5-25.5)	0.057
pRNFL *thickness, µm mean (SD)*	-	-	-
GCIPL *thickness, µm mean (SD)*	-	-	-
WBV, *mL mean (SD)*	1437.8 (94.9)	1422.1 (84.3)	0.637
DGMV, *mL mean (SD)*	44.3(3.1)	45.2 (4.2)	0.425
CGMV, *mL mean (SD)*	801.9 (42.7)	801.9 (42.7)	0.604
T2LV, *mL mean (SD)*	12.9 (8.1)	13.1 (11.9)	0.943

Total MS-SMART trial participants at UCL n= 176 and at Edinburgh n=93.
Did not consent OCT sub-study n=9
Consented OCT sub-study n=260

Baseline characteristics compared using t-tests or Wilcoxon rank sum tests as appropriate.

9HPT is calculated as the average of the mean of the two attempts with each hand. OCT and SLCVA measures are calculated as right- and left-eye means.
^Median (interquartile range).
Brain volumes are normalised with SIENAX.
9HPT = 9-hole peg test, CGMV = cortical grey matter volume, DGMV = deep grey matter volume, EDSS = expanded disability status scale, GCIPL = ganglion cell inner-plexiform layer, IQR: interquartile range, pRNFL = peripapillary retinal nerve fibre layer, SD: standard deviation. SDMT = symbol digit modalities test, SLCVA = Sloan low contrast visual acuity, T25FW = timed 25-foot walk, T2LV = T2 lesion volume, WBV = whole-brain volume.

Supplementary Table 2. Baseline characteristics of patients at the two study sites (UCL and Edinburgh).

	UCL N= 138	Edinburgh N= 74	P value
Age, *years mean (SD)*	54.2 (6.9)	54.8 (7.2)	0.557
Female sex, *no (%)*	96 (69.6%)	53 (71.6%)	-
Disease duration, *years mean (SD)*	22.05 (9.4)	22.8 (10.5)	0.607
Progression duration, *years mean (SD)*	8.2 (5.6)	8.2 (6.2)	0.96
EDSS, *score^ median (IQR)*	6.0 (5.5–6.5)	6.0 (6.0–6.5)	**0.010**
9HPT*, sec mean (SD)*	42.9 (65.5)	33.9 (15.0)	0.125
T25FW, *sec mean (SD)*	18.9 (26.0)	17.6 (15.7)	0.653
SDMT, *no. of correct answers median (IQR)*	49.5 (42-54)	43 (35-52)	**0.008**
SLCVA 2.5%, *no. of correct answers median (IQR)*	18.5 (10-29)	17.75 (9.25-25.5)	0.339

***pRNFL** thickness, μm mean (SD)*	84.4 (13.2)	81.3 (13.8)	0.113
***GCIPL** thickness, μm mean (SD)*	76.5 (13.6)	72.8 (14.2)	0.063
***WBV,** mL mean (SD)*	1421.3 (79.1)	1437.4 (66.6)	0.12
***DGMV,** mL mean (SD)*	45.7 (3.9)	44.5 (3.3)	**0.021**
***CGMV,** mL mean (SD)*	795.5 (41.9)	800.9 (34.3)	0.312
***T2LV,** mL mean (SD)*	11.05 (9.1)	13.4 (14.7)	0.217

Baseline characteristics compared using t-tests or Wilcoxon rank sum tests as appropriate.

9HPT is calculated as the average of the mean of the two attempts with each hand. OCT and SLCVA measures are calculated as right- and left-eye means.
^Median (interquartile range).
Brain volumes are normalised with SIENAX.
9HPT = 9-hole peg test, CGMV = cortical grey matter volume, DGMV = deep grey matter volume, EDSS = expanded disability status scale, GCIPL = ganglion cell inner-plexiform layer, IQR: interquartile range, pRNFL = peripapillary retinal nerve fibre layer, SD: standard deviation. SDMT = symbol digit modalities test, SLCVA = Sloan low contrast visual acuity, T25FW = timed 25-foot walk, T2LV = T2 lesion volume, WBV = whole-brain volume.

Supplementary Table 3. Baseline characteristics of subjects not included in analysis for any reasons versus subjects analysed at baseline

	**Subjects not included at baseline n=57**	**Included at baseline n=212**	**P value**
***Age,** years mean (SD)*	54.5 (8.5)	54.4 (7)	0.900
***Female sex,** no (%)*	42 (73.7)	149 (63)	-
***Disease duration,** years mean (SD)*	22.2 (9.4)	22.3 (9.8)	0.969
***Progression duration,** years mean (SD)*	9.0 (6.2)	8.2 (5.8)	0.426
***EDSS,** score^ median (IQR)*	6.0 (6.0–6.5)	6.0 (5.5–6.5)	0.072
***9HPT**, sec mean (SD)*	68.3 (98)	39.8 (53.7)	**<0.001**
***T25FW,** sec mean (SD)*	26.5 (40.7)	18.5 (22.9)	0.158
***SDMT,** no. of correct answers median (IQR)*	42 (28-49)	48 (39-53)	**<0.001**
***SLCVA 2.5%,** no. of correct answers median (IQR)*	11 (3-17)	18 (10-28.125)	**<0.001**
***pRNFL** thickness, μm mean (SD)*	-	-	-
***GCIPL** thickness, μm mean (SD)*	-	-	-
***WBV,** mL mean (SD)*	1406.6 (112.3)	1426.9 (75.1)	0.201
***DGMV,** mL mean (SD)*	44.7 (5.3)	45.2 (3.8)	0.491
***CGMV,** mL mean (SD)*	783.2 (56.2)	797.4 (39.4)	0.078
***T2LV,** mL mean (SD)*	17.6 (12.3)	11.9 (11.4)	**0.002**

Subjects not included at baseline n=57 (n=9 subjects did not consent; n=2 subjects excluded as we could not estimate history of optic neuritis; 46 excluded for eye disease, severe refractive errors, other reason (also reported in Figure 1).

Baseline characteristics compared using t-tests or Wilcoxon rank sum tests as appropriate.

9HPT is calculated as the average of the mean of the two attempts with each hand. OCT and SLCVA measures are calculated as right- and left-eye means.
^Median (interquartile range).
Brain volumes are normalised with SIENAX.
9HPT = 9-hole peg test, CGMV = cortical grey matter volume, DGMV = deep grey matter volume, EDSS = expanded disability status scale, GCIPL = ganglion cell inner-plexiform layer, IQR: interquartile range, pRNFL = peripapillary retinal nerve fibre layer, SD: standard deviation. SDMT = symbol digit modalities test, SLCVA = Sloan low contrast visual acuity, T25FW = timed 25-foot walk, T2LV = T2 lesion volume, WBV = whole-brain volume.

Supplementary Table 4. pRNFL rate of change for all subjects

Group	rate	Standard error	Lower CI	Upper CI	P value
Overall	-0.8305050	0.1060385	-1.039138	-0.6218718	7.399937e-14
NON	-0.9688753	0.1066039	-1.178621	-0.7591296	1.146550e-17
ON	-0.6921347	0.1769675	-1.040322	-0.3439471	1.125736e-04

Supplementary Table 5. GCIPL rate of change for all subjects

Eye Group	rate	Standard error	Lower CI	Upper CI	P value
Overall	-0.3716247	0.08290540	-0.5347371	-0.208512274	1.030936e-05
NON	-0.4922726	0.08241912	-0.6544283	-0.330116970	6.238899e-09
ON	-0.2509767	0.12858783	-0.5039671	0.002013655	5.184040e-02

Supplementary Table 6. GCIPL rate of change Edinburgh site

Eye Group	rate	Standard error	Lower CI	Upper CI	P value
Overall (n=94)	-0.7641149	0.2122729	-1.186030	-0.3421994	0.0005289943
NON (=60)	-0.7046296	0.2022596	-1.106642	-0.3026168	0.0007762646
ON (=34)	-0.8236002	0.3286565	-1.476841	-0.1703599	0.0140727299

Supplementary Table 7. GCIPL rate of change UCL site

Eye Group	rate	Standard error	Lower CI	Upper CI	P value
Overall (n=231)	-0.23187410	0.07657466	-0.3827551	-0.08099313	2.743129e-03
NON (n=131)	-0.38944799	0.07766934	-0.5424859	-0.23641008	1.066820e-06
ON (n= 100)	-0.07430022	0.12161602	-0.3139297	0.16532924	5.418446e-01
